# Temperature Differentially Influences the Capacity of *Trichoderma* Species to Induce Plant Defense Responses in Tomato Against Insect Pests

**DOI:** 10.3389/fpls.2021.678830

**Published:** 2021-06-09

**Authors:** Ilaria Di Lelio, Mariangela Coppola, Ernesto Comite, Donata Molisso, Matteo Lorito, Sheridan Lois Woo, Francesco Pennacchio, Rosa Rao, Maria Cristina Digilio

**Affiliations:** ^1^Department of Agricultural Sciences, University of Naples Federico II, Naples, Italy; ^2^Task Force on Microbiome Studies, University of Naples Federico II, Naples, Italy; ^3^Interuniversity Center for Studies on Bioinspired Agro-Environmental Technology (BAT Center), University of Naples Federico II, Naples, Italy; ^4^Department of Pharmacy, University of Naples Federico II, Naples, Italy

**Keywords:** induced systemic resistance, defense genes, gene expression analysis, *Macrosiphum euphorbiae*, *Spodoptera littoralis*, biological control

## Abstract

Species of the ecological opportunistic, avirulent fungus, *Trichoderma* are widely used in agriculture for their ability to protect crops from the attack of pathogenic fungi and for plant growth promotion activity. Recently, it has been shown that they may also have complementary properties that enhance plant defense barriers against insects. However, the use of these fungi is somewhat undermined by their variable level of biocontrol activity, which is influenced by environmental conditions. Understanding the source of this variability is essential for its profitable and wide use in plant protection. Here, we focus on the impact of temperature on *Trichoderma afroharzianum* T22, *Trichoderma atroviride* P1, and the defense response induced in tomato by insects. The *in vitro* development of these two strains was differentially influenced by temperature, and the observed pattern was consistent with temperature-dependent levels of resistance induced by them in tomato plants against the aphid, *Macrosiphum euphorbiae*, and the noctuid moth, *Spodoptera littoralis*. Tomato plants treated with *T. afroharzianum* T22 exhibited enhanced resistance toward both insect pests at 25°C, while *T. atroviride* P1 proved to be more effective at 20°C. The comparison of plant transcriptomic profiles generated by the two *Trichoderma* species allowed the identification of specific defense genes involved in the observed response, and a selected group was used to assess, by real-time quantitative reverse transcription PCR (qRT-PCR), the differential gene expression in *Trichoderma*-treated tomato plants subjected to the two temperature regimens that significantly affected fungal biological performance. These results will help pave the way toward a rational selection of the most suitable *Trichoderma* isolates for field applications, in order to best face the challenges imposed by local environmental conditions and by extreme climatic shifts due to global warming.

## Introduction

The need to reduce the use of chemical pesticides in agriculture has promoted the development of bio-based strategies of plant protection, exploiting beneficial organisms, and the ecological services they provide. There are various biological products available on the market used for disease and pest control, as well as plant biostimulants and fertilizer/soil enhancers that are based on plant beneficial microbes, such as bacteria [*Bacillus, Pseudomonas* (Ferreira et al., 1991; Walsh et al., 2001)] or fungi [*Trichoderma*, mycorrhizae*, Beauveria* (Castillo Lopez and Sword, 2015; Rouphael et al., 2015; Russo et al., 2015; Sinno et al., 2020)]. Numerous *Trichoderma* strains are the key components of commercially available microbial biofungicides (Harman et al., 2004; Woo et al., 2014; Alfiky and Weisskopf, 2021; Ferreira and Musumeci, 2021; Poveda, 2021). Among the 377 *Trichoderma* species identified, only 20–30 species are found in soils as ecological opportunists saprophytes, associated with the rhizosphere, avirulent to plants, that may be useful in agriculture (Druzhinina et al., 2011; Cai and Druzhinina, 2021). It cannot be generalized that all *Trichoderma* are extensive root colonizers or that they are endophytes (Howell, 2003; Harman et al., 2004). For example, some isolates exhibit localized penetration of plant tissues, whereas others are rhizosphere competent, and are able to colonize the roots by growing in the epidermis and outer cortex of the tissues (Harman, 2000; Harman et al., 2004; Hermosa et al., 2012; Lace et al., 2015). Many *Trichoderma* are indeed free-living microorganisms in the soil rhizosphere and have limited contact with the plant; however, by releasing microbial compounds recognized by the plant, they are still able to interact in a molecular cross-talk that influences the plant defense response (Lorito et al., 2010). Some of these antagonists of phytopathogens, not only contribute to biocontrol, but also provide well-recognized positive effects to the plant, such as improved vegetative-root growth, development, and yield; they also enhance nutrient availability and uptake by the plant (Vinale et al., 2008). For example, T. *afroharzianum* (ex-T. *harzianum*; Cai and Druzhinina, 2021) strain T22, the active component of a commercial biofungicide product, was also found to improve the plant growth of important horticultural crops, such as lettuce, tomatoes, peppers, ornamentals, and woody crops, and to prevent diseases, both under greenhouse and field conditions (Harman et al., 2004; Lorito et al., 2010; Woo et al., 2014).

Different *Trichoderma* species or strains can have a diverse impact on different crop species, or even genotypes of the same crop, as observed in tomato in which *T. afroharzianum* T22 and *Trichoderma atroviride* P1, diversely affected plant growth and resistance against *Botrytis cinerea*, depending upon the tomato genotype (Tucci et al., 2011). Furthermore, the crop genotype was found to significantly influence the colonization by different *Trichoderma* strains in the rhizosphere of lentils (Bazghaleh et al., 2020). *Trichoderma* may promote plant endogenous defenses against biotic (phytopathogenic fungi) and abiotic stress factors by induced local or systemic resistance (ISR), similar to those activated by plant growth promoting rhizobacteria (PGPR) that result in the priming of the plant to subsequent attacks by pathogens or other parasites (Harman et al., 2004; Lorito et al., 2010; Hermosa et al., 2013; Conrath et al., 2015; Martínez-Medina et al., 2017; Adnan et al., 2019). *Trichoderma* is also capable of stimulating an ISR plant defense response against nematodes, modulated by a cross-talk between salicylic acid (SA) and jasmonic acid (JA) signaling pathways (Martínez-Medina et al., 2017). Furthermore, it was observed that plants exposed to *Trichoderma* root colonization were more resistant to pest insects, such as aphids (Coppola et al., 2019a), thrips (Muvea et al., 2014), caterpillars (Contreras-Cornejo et al., 2018; Coppola et al., 2019b), and nematodes (Poveda et al., 2020). Indirect defense barriers against insects are also induced in plants colonized by *Trichoderma*, whereby the plant releases compounds that attract the parasitoids (Coppola et al., 2017, 2019a) and/or predators (Battaglia et al., 2013) of the pest insects that attack the plants.

One of the major problems associated with the use of beneficial microbes in agriculture is the variability of their effects on target organisms, which can be affected by environmental conditions. Indeed, the selection of appropriate *Trichoderma* for applications in agriculture depends not only on the targeted use, that is, the biological control of pathogens or pests or the use as biostimulants, but also on the ecological adaptability of the strains to diverse environments (i.e., soils of different properties—structure, pH, and organic matter), availability of water and nutrients, climatic conditions and to crops (i.e., species or genotypes) that may influence their efficacy in the field (Hjeljord and Tronsmo, 1998). Many authors reported that temperature has an effect on spore germination, hyphal growth, and colonization of the biocontrol agent that consequently influences the competitive and antagonistic capabilities of the *Trichoderma* strain, due to its differential interaction with the target fungal pathogen (Tronsmo and Dennis, 1978; Mukherjee and Raghu, 1997; Kredics et al., 2003). Furthermore, the disparity in the growth and development of this plant beneficial microbe at diverse temperatures can have a key role in the *Trichoderma*-host plant interaction, the establishment of the molecular cross-talk that underpins the activation of the plant defense response involving induced resistance to pathogen infection or parasite infestation. The study of the variability in direct and indirect biocontrol efficacy that is dependent on temperature is essential to enhance the action and the application of these microbial agents.

To fill this research gap, the objective of this study is to investigate how temperature impacts the biological performance of two different *Trichoderma* strains (*T. afroharzianum* T22 and *T. atroviride* P1) and the consequential effects they have on transcriptional reprogramming in treated tomato plants to activate defense barriers against insects. Our results will help pave the way toward a rational selection of the most suitable strains of *Trichoderma* for field applications, in order to better face the challenges posed by local environmental settings and by conditions resulting from sudden extreme changes due to global warming.

## Materials and Methods

### Fungal Isolates

*Trichoderma afroharzianum* strain T22, hereafter indicated as T22, was isolated from the commercial biofungicide product, Trianum (kindly provided by Koppert Biological Systems, Rotterdam, the Netherlands). *Trichoderma atroviride* strain P1, hereafter indicated as P1 (ATCC 74058) was obtained from the microbial collection of the Department of Agricultural Sciences, University of Naples at Portici, Italy. It is a strain that was isolated from wood chips and selected as an effective biological control agent against foliar and post-harvest pathogens, such as *B. cinerea*, and for use in cold storage (Tronsmo, 1991).

Both fungi were maintained on potato dextrose agar (PDA; HiMedia) at room temperature (25°C) and sub-cultured regularly. Conidia were collected from the surface of sporulating fungal cultures (5–7 days), in sterile distilled water and adjusted to a concentration of 10^7^ sp ml^−1^ by using a hemocytometer (Coppola et al., 2019b).

### Plant Material and *Trichoderma* Treatment

A seed-coating treatment was conducted on surface-sterilized seeds of *Solanum lycopersicum* cv “Dwarf San Marzano” following the protocol of Coppola et al. (2019a). Treated seeds were planted in 60-well planting trays containing sterilized soil (Floragard, Universal Potting Soil, Oldenburg, Germany), then 7–10 days after emergence, plant plugs were transplanted to 10 cm diameter vases and grown at different temperature conditions of 20 ± 1°C or 25 ± 1°C, photoperiod 16: 8 h light/dark. Under these two temperatures, experimental plants attained the size for their use in the insect bioassays after 7 weeks at 20°C and 5 weeks at 25°C.

### Insect Rearing

*Spodoptera littoralis* is permanently lab-reared at the Department of Agricultural Sciences and derives from a population collected on flower crops in Agro-Pontino (Latina, Italy). The larvae were reared on an artificial diet (41.4 g/l wheat germ, 59.2 g/l brewer's yeast, 165 g/l corn meal, 5.9 g/l ascorbic acid, 1.53 g/l benzoic acid, 1.8 g/l methyl-4-hydroxybenzoate, and 29.6 g/l agar), as previously described (Di Lelio et al., 2014), at 25 ± 1°C, 70 ± 5% RH, and photoperiod of 16:8 h light/dark.

The aphid *Macrosiphum euphorbiae* was collected on tomato crops (Battipaglia, Salerno, Italy) and is permanently reared on tomato plants (*S. lycopersicum*, cultivar Dwarf San Marzano) placed in anti-aphid netcages, in a greenhouse at 20 ± 2°C, 70 ± 10% RH, and photoperiod of 16:8 h light/dark.

### Fungal Development at Different Temperatures

The two *Trichoderma* (strains T22 and P1) were cultured in 90 mm Petri plates containing PDA, incubated at 25°C, for 3 days, in the dark. A fungal plug (5 mm) was transferred to the center of new PDA plates and both *Trichoderma* inoculated cultures were incubated in the same controlled growth conditions as the plants reared for the insect bioassays, at temperatures of 20 and 25°C. Four plates were inoculated for each fungal strain, for each of the two incubation temperatures (4 × 2 = 8 cultures per strain); and the experiment was repeated two times. The radial growth of the fungal mycelia was measured at 24, 48, and 72 h.

### Fungal Colony-Forming Units in Soil

At the end of insect bioassays (30 days at 20°C and 32 days at 25°C), the samples were collected from the soil in the vases of potted tomato plants at the first flowering stage, to quantify the number of fungal colony-forming units (CFUs) and confirm the presence of living *Trichoderma* after the seed treatments. A mixture of 10 g of soil containing plant roots was added to 90 ml Ringer solution (Sigma-Aldrich, Milan, Italy), containing 0.162 g of sodium pyrophosphate, then placed in agitation for 30 min on a “tilting top” mixer (SSL1, Stuart, Staffordshire, United Kingdom), and this soil suspension was used to prepare a serial dilution from 10^−3^ to 10^−7^ with sterile water. A 100 μl aliquot of the soil suspension from each dilution series was transferred to plates containing *Trichoderma* selective media agar (TSM; HiMedia Laboratories LLC, PA, United States) augmented with Igepal, then distributed uniformly on the substrate surface with an L-spreader, and incubated at 25°C. After 5 days, the number of *Trichoderma* colonies was counted to determine the abundance of fungi present in the soil rhizosphere.

### *Spodoptera littoralis* Bioassay

The larval feeding bioassay was performed at 25 ± 1°C and 70 ± 5% RH, and photoperiod of 16:8 h light/dark, in 4-wells plastic rearing trays (RT32W, Frontier Agricultural Sciences, Pitman, NJ, United States). In each well, 3 ml of 1.5% agar-agar (w/v) were dispensed, in order to keep the tomato leaves turgid in a moist environment, and the rearing wells were closed by perforated plastic lids (RTCV4, Frontier Agricultural Sciences, Pitman, NJ, United States). Groups of 25 newly hatched larvae were isolated into a single well (for a total of 400 larvae/treatment) and allowed to feed on sub-apical tomato leaf disks of 6 week-old plants. Then, newly molted third instar larvae were singly transferred into the wells of a new tray, prepared as above, and were offered fresh leaf disks daily, obtained from sub-apical leaves of tomato plants reared at 20°C or at 25°C. For each treatment, 32 larvae and 60 tomato plants were used.

On a daily basis, the following parameters were recorded: survival of the larvae and their weight, the number of days to attain the pupal stage, the weight of the pupae, the rate of adult emergence, and longevity. To assess the fertility of the emerged adults and the viability of their eggs, each female moth was fed soon after emergence with water/honey solution (50%) and allowed to mate with two males for 24 h, then isolated in a box (40 × 30 × 20) to assess the number of eggs laid daily and their rate of hatching.

### *Macrosiphum euphorbiae* Bioassay

To assess the effects of plant defense barriers induced by *Trichoderma* on sucking insects, a bioassay on aphids was carried out. Briefly, five apterous adult aphids were gently transferred onto a single plant using a paintbrush. After 24 h, the adult aphids were removed and only five nymphs of the newly laid progeny were left on the plant. Aphid survival was recorded daily, until the survival of the last aphid. The laid nymphs were counted and removed every day until the end of reproductive activity. The bioassay was conducted in a glasshouse under controlled climatic conditions, at the temperature of 20 or 25°C in separate experimental settings, kept at 70 ± 10% RH and photoperiod of 16:8 h light/dark. A total of 11 replicates (a single tomato plant constituted a replicate) were carried out for each experimental treatment (T22, P1, control).

### Gene Expression Analysis

Expression levels of defense-related genes were quantified by real-time PCR (RT-PCR). Fully expanded leaves from plants grown at 20 and 25°C were collected at seven and 5 weeks after sowing, respectively, and immediately frozen in liquid nitrogen.

The isolation of total RNA and the synthesis of the first strand of cDNA were performed according to standard procedures, as already described (Corrado et al., 2012). Expression analysis was carried out using two technical replicates for each of the three biological replicates per sample. Relative quantification of gene expression was carried out using the 2^−ΔΔCt^ method (Livak and Schmittgen, 2001). Student's *t*-test was used to compare the relative quantification of transcripts in treated samples compared to the untreated controls, used as calibrator. The housekeeping gene EF-1α was used as an endogenous reference gene for the normalization of the expression levels of the target genes. Primers and their main features are reported in Supplementary Table 1.

The differentially expressed genes (DEGs) originating from two public datasets reporting the transcriptomic profiles of tomato plants modulated by *T. atroviride* P1 (Coppola et al., 2019a) or by *T. afroharzianum* T22 treatments (Coppola et al., 2019b), generated from tomato plants grown at 20°C, were compared by Venny (Oliveros, 2007). DEGs were mapped to MapMan bins for data visualization and pathway analysis (version 3.6.0). To this end, the tomato MapMan ontologies (http://www.gomapman.org/export/current/mapman/sly_SL2.40_ITAG2.3_2015-01-09_mapping.txt.tgz) were retrieved from the GO MapMan web resource and imported in the MapMan tool.

### Statistical Analysis

Survival curves of *S. littoralis* and *M. euphorbiae* were compared by using Kaplan–Meier and Log-rank analysis. Unpaired Student's *t*-test was used for pairwise comparisons of the means, and one-way ANOVA test was used when more than two groups were involved. Normality of data was checked with Shapiro–Wilk test and Kolmogorov–Smirnov test, while homoscedasticity was tested with Levene's test and Barlett's test. When significant effects were observed (*P* < 0.05), Bonferroni's *post-hoc* test was used. If one of the one-way ANOVA assumptions was not met, even after the transformation of the data, Kruskal–Wallis (non-parametric ANOVA) test was employed. Fisher's test was performed at *P* ≤ 0.05 to compare the mean mycelial growth of *Trichoderma* strains (MiniTab, Windows). Data were analyzed using Prism (GraphPad Software Inc. version 6.0b, San Diego, CA, United States).

## Results

### Fungal Development

**Figure 1:**
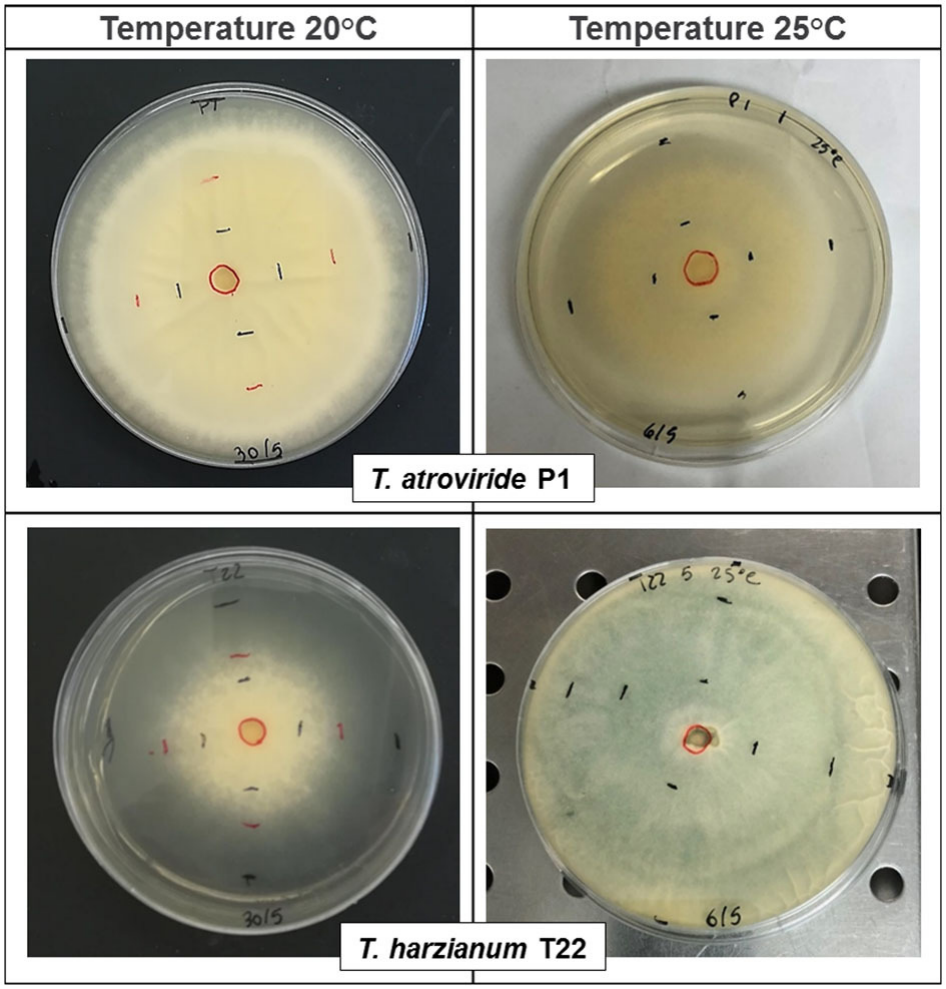
Effect of temperature, at 20°C (left) and 25°C (right), on the mycelial growth of *Trichoderma atroviride* P1 and *T. afroharzianum* T22 after 72 h. Measurements were conducted every 24 h, as indicated by the black/red markings delineating the periphery of the fungal colony. The fungi were cultured on potato dextrose agar (PDA) and maintained at a constant temperature of 20 ± 1°C or 25 ± 1°C and photoperiod of 16: 8 h light/dark.

*Trichoderma* strains, T22 and P1 showed differential growth at 20°C and 25°C in the *in vitro* plate cultures (Table 1, Figure 1). At 20°C, the mycelial growth of P1 was more rapid than that of T22 (Table 1), already significantly higher at the second day [one-way ANOVA: *F*_(1, 6)_ = 21.55; *P* = 0.004], and by day three, the difference was even more pronounced [one-way ANOVA: *F*_(1, 6)_ = 405.60; *P* < 0.0001]. At 72 h, the P1 mycelium was dense and completely covered the surface of the Petri dish (90 mm diameter), whereas the T22 culture was thin and required an additional day to cover the entire substrate (Figure 1 left, Table 1). At 25°C, the growth of the two strains was inversed, with T22 demonstrating a significantly faster growth than P1 at all sampling times [one-way ANOVA: 24 h *F*_(1, 6)_= 130.85; *P* < 0.0001; 48 h *F*_(1, 6)_ = 170.97; *P* < 0.0001; 72 h *F*_(1, 6)_ = 76.80; *P* < 0.0001], and its dense mycelium completely covered the plate by day three, whereas P1 required an additional day to attain the same dimension (Figure 1 right, Table 1). It can be noted that T22 had a faster mycelial growth rate than P1, in particular, when the fungal development was compared at the optimal temperature for each strain [one-way ANOVA: 24 h *F*_(3, 12)_ = 122.10; *P* < 0.0001; 48 h *F*_(3, 12)_ = 157.20; *P* < 0.0001; 72 h *F*_(3, 12)_ = 130.93; *P* < 0.0001) (Table 1).

**Table 1:** Effect of temperature (20°C and 25°C) on the mycelial growth (colony diameter in mm) of *Trichoderma* strains T22 and P1 cultured on PDA, measured at 24 h intervals.

		**Mycelial growth (mm) over time**		
**Temperature**	***Trichoderma***	**24 h**	**48 h**	**72 h**
20°C	T22	21.25 ± 1.26 c	41.50 ± 1.73 c	64.00 ± 2.58 c
20°C	P1	22.50 ± 1.92 c	47.50 ± 2.06 b	90.00 ± 0.00 a
25°C	T22	42.75 ± 1.71 a	75.00 ± 2.45 a	90.00 ± 0.00 a
25 °C	P1	27.00 ± 2.16 b	49.25 ± 2.99 b	74.00 ± 3.65 b

*Values are the means of four replicates ± SD. Different letters in the same column indicate significant differences within each sampling time, according to Fisher's test (P = 0.05)*.

### Fungi in Soil

**Figure 2:**
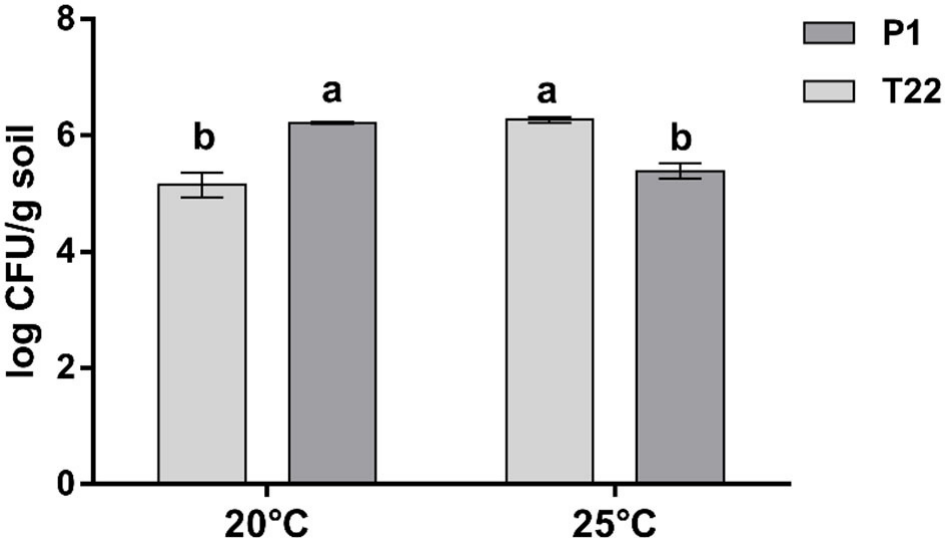
*Trichoderma* colonies in soil. Effect of temperature (20 and 25°C) on the number (means ± SD) of fungal colony-forming units (CFU) in soil samples obtained from the rhizosphere of tomato plants inoculated with *Trichoderma* strain T22 or P1, 5 days after inoculation on *Trichoderma* selective media (TSM). Values are the means of three replicates; bars with different letters indicate significant differences according to Fisher's test at *P* ≤ 0.05.

At 20°C, a significantly higher number of fungal colonies was obtained from soils of plants receiving the P1 seed treatment (Student's *t*-test: t = 9.88, *P* < 0.001, dF = 4), whereas at 25°C, a significantly higher number of CFUs was found in the soils from the T22 treated plants (Student's *t*- test: t = 14.632, *P* < 0.0001, dF = 4) (Figure 2). The CFU abundance in the soil of the *Trichoderma* strains at the two temperatures of incubation was indicative of the corresponding trend observed for the differential mycelial growth of P1 at 20°C and T22 at 25°C demonstrated in the *in vitro* tests.

### *Spodoptera littoralis* Bioassay

**Figure 3:**
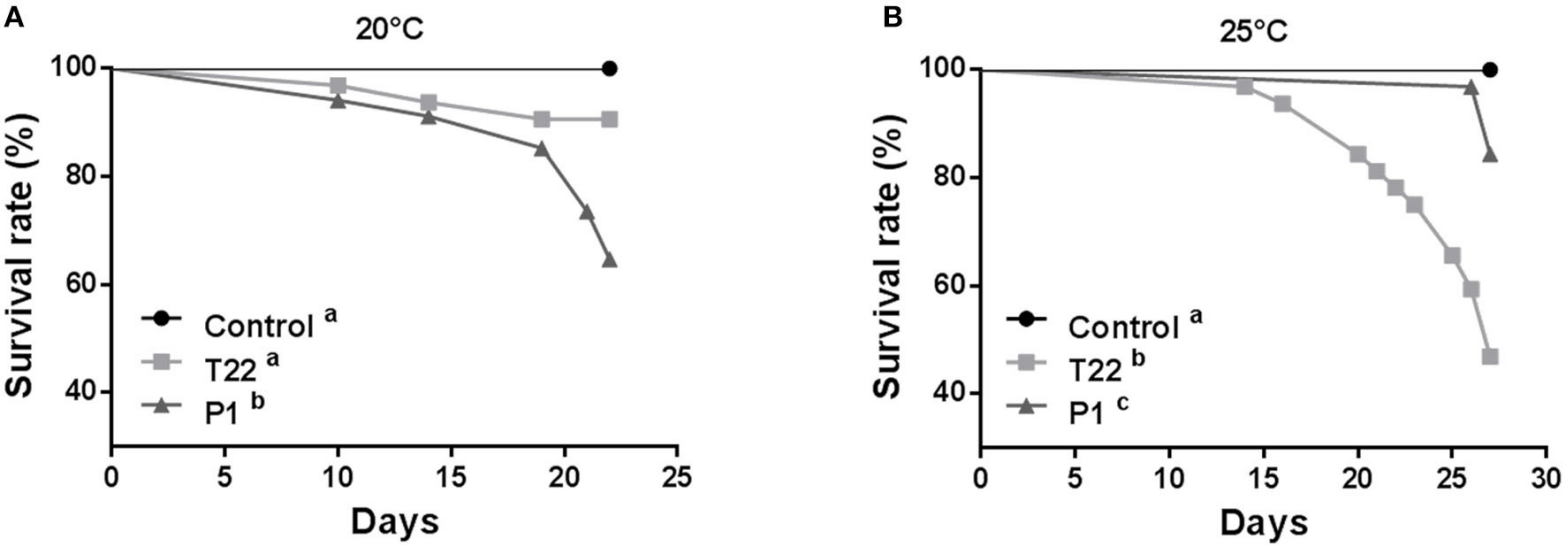
*Spodoptera littoralis* larvae survival when fed leaves from tomato plants inoculated with *Trichoderma strain* T22 or P1 then grown at 20°C **(A)** or 25°C **(B)**. At 20°C, **(A)** survival was significantly lower on T22-tomato with respect to P1- and Control- tomato, whereas, when plants were grown at 25°C, **(B)** larvae fed on T22- or P1-tomato showed higher mortality compared to Control. Different letters denote significant differences in the survival curves (Log-Rank test, *P* < 0.0001).

Since the two *Trichoderma* strains tested showed highly different performances at the two experimental temperatures considered, it was assessed whether the resulting different interaction with colonized plants at different temperatures can affect the activation of defense barriers against insects. At 20°C, *S. littoralis* showed the lowest fitness when reared on plants inoculated with P1 (Figure 3A). T22 and P1 treatments to tomato plants grown at 20°C significantly affected larval survival until pupation (Log-rank test: χ^2^ = 16.80; *P* = 0.0002; dF = 2). However, the survival rate of larvae fed on P1-tomato leaves was significantly lower, compared both to controls and T22-tomato plants (Log-rank test: P1 vs. control χ^2^ = 13.58; *P* = 0.0002; df = 1; P1 vs. T22 χ^2^ = 5.676; *P* = 0.0172; dF = 1), while no difference was registered between the latter two (Log-rank test: χ^2^ = 3.098; *P* = 0.0784; dF = 1) (Figure 3A).

Conversely, at 25°C, the strain most negatively affecting *S. littoralis* fitness was T22 (Figure 3B). Under these experimental conditions, both *Trichoderma* strains negatively affected larval survival (Log-rank test: χ^2^ = 31.55; *P* < 0.0001; dF = 2), but, unlike what observed at the lower temperature, T22 had a significantly higher negative impact on larval survival compared both to the control (Log-rank test: χ^2^ = 23.12; *P* < 0.0001; dF = 1), and to the P1 treatment (Log-rank test: χ^2^ = 11.90; *P* = 0.0006; dF = 1) (Figure 3B).

The pattern of reduced survival across larval-pupal development, as affected by temperature, was observed also for the weight of larvae and pupae and for the time required to attain the pupal stage (Supplementary Figures 1, 2, and Supplementary Table 2).

**Figure 4:**
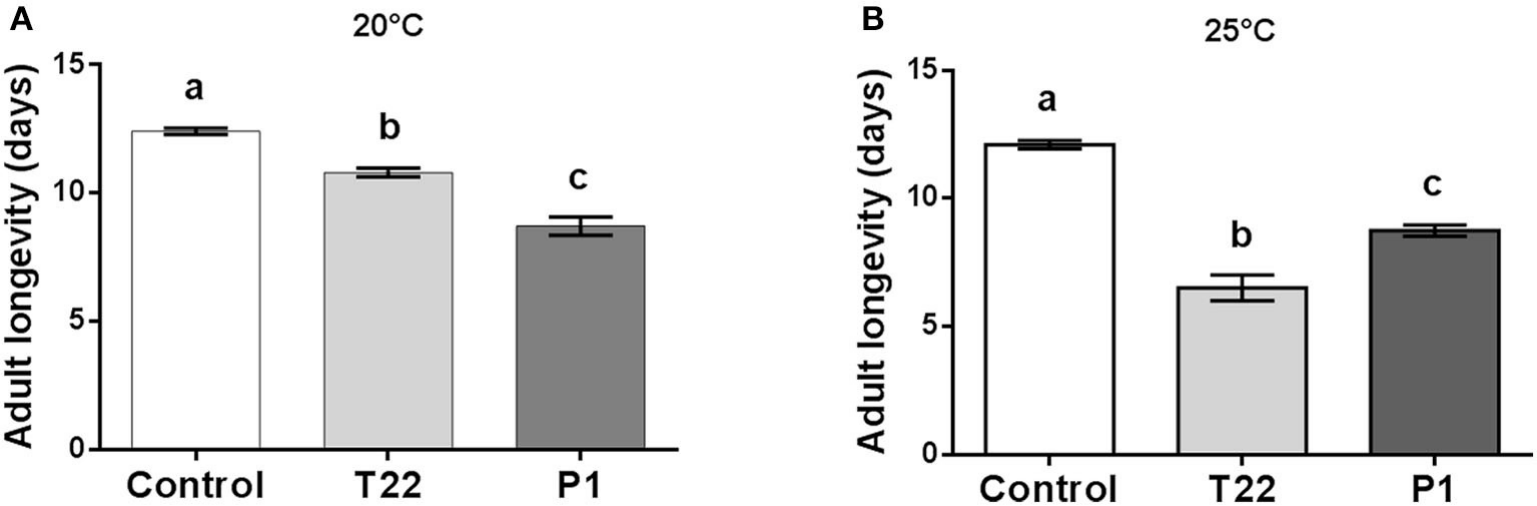
*Spodoptera littoralis* adult longevity. Adults emerging from larvae fed on T22- and P1-tomato leaf disks showed a significantly lower longevity compared to Control adults. At 20°C, **(A)** P1-tomato had the highest negative impact on longevity, whereas at 25°C, the reverse was observed, with T22 showing a significantly higher negative impact on longevity. The values are means ± SD. Mean values denoted with different letters are significantly different (one-way ANOVA **(A)** or Kruskal–Wallis test **(B)** (*P* < 0.05).

Tomato plants colonized by *Trichoderma* had a negative impact on *Spodoptera* adult longevity, which was significantly higher when they were fed as larvae on P1-tomato at 20°C [one-way ANOVA: *F*_(2, 65)_ = 79.84; *P* < 0.0001] (Figure 4A), while, at 25°C, T22-tomato resulted as the food source most detrimental for adult longevity [one-way ANOVA: *F*_(2, 59)_ = 126.1; *P* < 0.0001] (Figure 4B).

**Figure 5:**
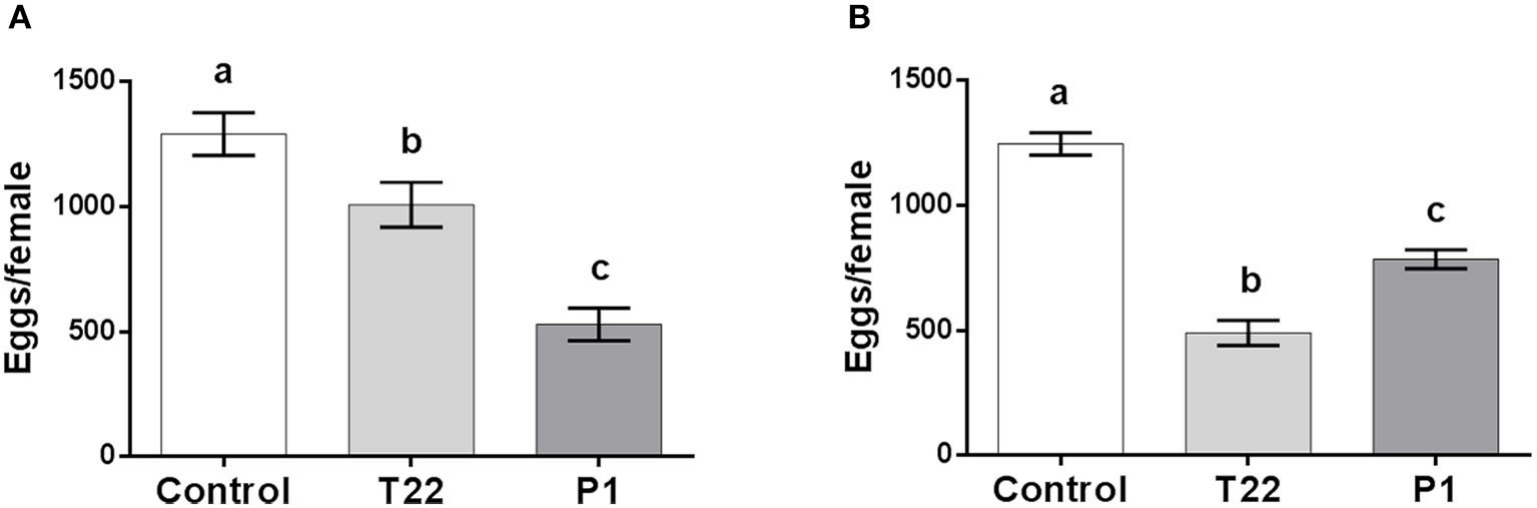
*Spodoptera littoralis* fertility. Adults emerging from larvae fed on T22- and P1-tomato leaf showed lower fertility compared to adults. At 20°C, **(A)** the lower performance was observed on P1-tomato, whereas at 25°C, **(B)** fertility was significantly poorer on T22-tomato. The values are means ± SD. Mean values denoted with different letters are significantly different (one-way ANOVA test, *P* < 0.0001).

Adult fertility was similarly affected, with P1 resulting most detrimental at 20°C [one-way ANOVA: *F*_(2, 24)_ = 13.59; *P* < 0.0001] (Figure 5A), while at 25°C, the reverse occurred, with females deriving from larvae fed on T22-tomato leaves producing less eggs [one-way ANOVA: *F*_(2, 26)_ = 61.86; *P* < 0.0001] (Figure 5B).

### *Macrosiphum euphorbiae* Bioassay

*M*. *euphorbiae* showed a lower survival on tomato plants inoculated with *T. atroviride* P1 and incubated at 20°C, while at 25°C, the reverse was observed, with *T. afroharzianum* T22 showing a higher negative impact on the insect.

**Figure 6:**
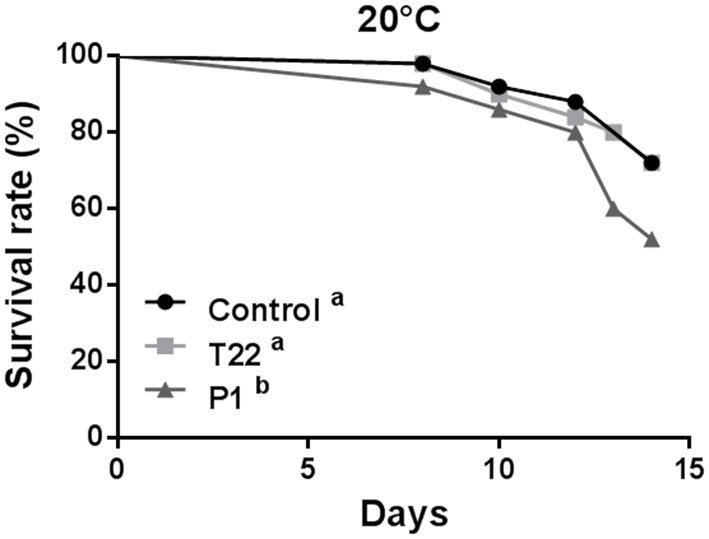
Aphid survival rate at 20°C. *Macrosiphum euphorbiae* reared on P1-tomato showed higher mortality compared to aphids reared on T22- and Control-tomato plants. Different letters indicate a significant difference (Log-rank test, *P* < 0.001).

At 20°C, aphid survival was affected by feeding on plants receiving the *Trichoderma* treatment (Log-rank test: χ^2^ = 6.740; *P* = 0.0344; dF = 2), showing a significant reduction on P1-tomato plants compared to control (Log-rank test: χ^2^ = 4.898; *P* = 0.0269; dF =1), or to T22-tomato (Log-rank test: χ^2^ = 3.666; *P* = 0.0555; dF = 1), which did not significantly differ between them (Log-rank test: χ^2^ = 0.1031; *P*= 0.7481; dF = 1) (Figure 6).

**Figure 7:**
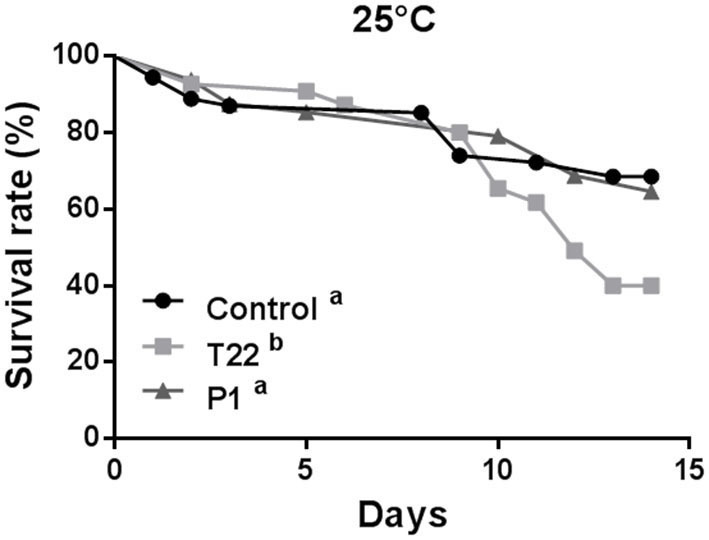
Aphid survival rate at 25°C. *Macrosiphum euphorbiae* reared on T22-tomato showed higher mortality compared to aphids on P1- and Control-tomato plants. Different letters indicate a significant difference (Log-rank test, *P* < 0.001).

At 25°C, aphid survival was affected by *Trichoderma-*treated plants (Log-rank test: χ^2^ = 15.68; *P* = 0.0004; dF = 2), but, conversely to what observed at 20°C, survival rates registered on T22-tomato plants were significantly lower than those induced by both P1-plants (Log-rank test: χ^2^ = 11.92; *P* = 0.0006; dF = 1) and controls (Log-rank test: χ^2^ = 11.45; *P* = 0.0007; dF = 1), which did not differ between them (Log-rank test: χ^2^ = 0.1273; *P* = 0.72; dF = 1) (Figure 7).

**Figure 8:**
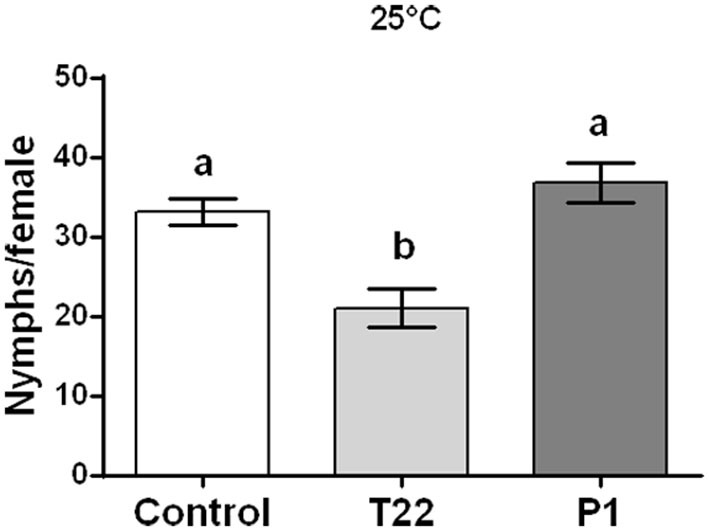
Aphid fertility on tomato plants grown at 25°C. Offspring of *Macrosiphum euphorbiae* reared for their whole lifespan on T22-, P1-, and Control-tomato plants. Data represent mean ± SE. Different letters indicate a significant difference (one-way ANOVA test, *P* < 0.05).

Aphid fertility was not influenced by *Trichoderma* treatments when plants were reared at 20°C [one-way ANOVA: *F*_(2, 23)_ = 2.023; *P* < 0.1551; data not shown], while a significant reduction was induced at 25°C by the T22-tomato treatment [one-way ANOVA: *F*_(2, 29)_ = 3.447; *P* < 0.0454] (Figure 8).

### *In silico* Comparison of Plant Transcriptomic Profiles Affected by *T. atroviride* P1 and *T. afroharzianum* T22

To assess the impact of the two *Trichoderma* species on the main defense-related pathways, we compared the transcriptomic profiles of tomato plants, as previously analyzed in precedent studies: tomato as affected by treatments with *T. atroviride* P1 (Coppola et al., 2019a) and *T. afroharzianum* T22 grown at 20°C (Coppola et al., 2019b).

**Figure 9:**
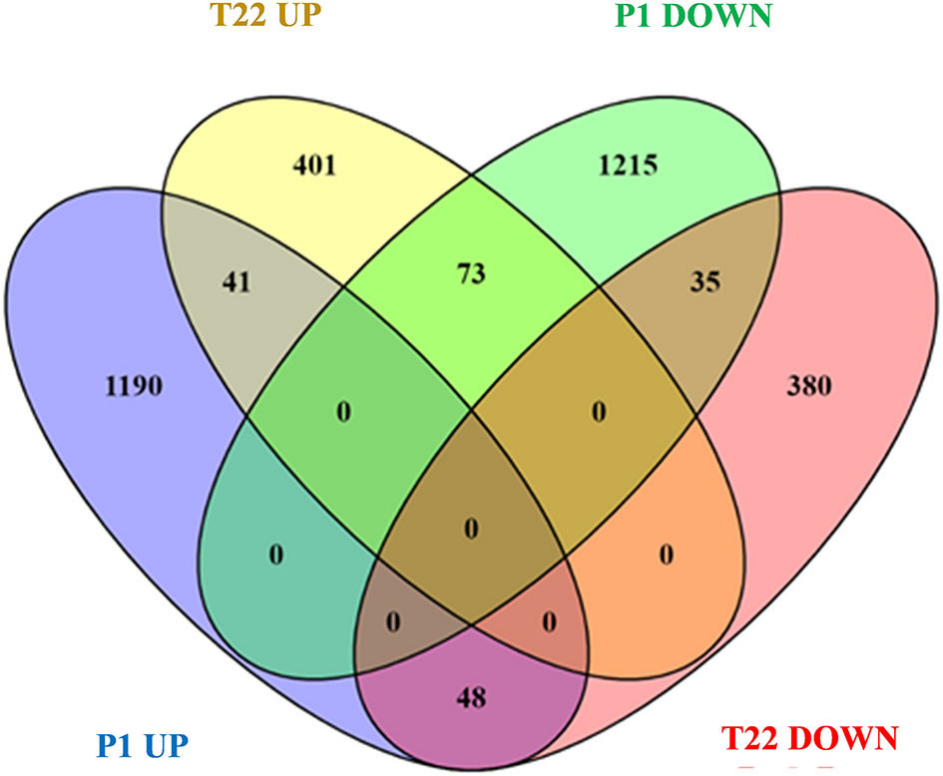
Venn diagram visualization of differentially regulated genes (DEGs) in P1 and T22 treated plants at 20°C. DEGs of tomato plants inoculated with *T. atroviride P1* (P1) and tomato plants inoculated with *T. afroharzianum* T22 (T22).

A Venn diagram representation of the *in silico* comparison of the two datasets (Figure 9) showed a common effect of the two fungi on gene expression, which shared 41 upregulated and 35 downregulated genes (Supplementary Tables 3A,B). Common genes induced by the tomato-*Trichoderma* interaction included genes coding for several classes of transcription factors, most of them associated with ethylene signaling (Table 2): ethylene-responsive transcription factor, TINY, ethylene-responsive transcription factor, MYB transcription factor 31, and AP2/EREBP transcription factor. In addition, several genes involved in tomato defense were induced by P1 or T22 seed treatments, such as polyphenol oxidase precursor, late embryogenesis abundant protein, heat-shock protein, purple acid phosphatase 17, metallocarboxypeptidase inhibitor (Supplementary Table 3A).

**Table 2:** Common DEGs of datasets coding for transcription factors in tomato treated with *Trichoderma* strains P1 or T22 and grown at 20°C.

**Gene ID**	**P1 FC**	**T22 FC**	**Description**
Solyc06g066540.1	2.93	1.47	Ethylene-responsive transcription factor TINY
Solyc01g090340.2	2.77	1.24	Ethylene-responsive transcription factor
Solyc03g116100.3	1.63	1.12	MYB transcription factor 31
Solyc08g082210.3	1.53	1.25	AP2/EREBP transcription factor
Solyc08g066660.1	1.23	1.19	Ethylene-responsive transcription factor TINY

Interestingly, both P1 and T22 treatments downregulated the genes associated with the cell wall, such as expansin-like protein, wall-associated receptor kinase-like 20, pectinesterase, and xyloglucan endotransglucosylase/hydrolase (Supplementary Table 3B).

A remarkable opposing effect on tomato transcriptomic profiles determined by the two different species of *Trichoderma* was evident for 73 genes, which were downregulated in P1 and upregulated in T22 samples (Figure 9; Supplementary Table 3C), and for 48 genes with opposite signs in the available datasets (Figure 9; Supplementary Table 3D).

Table 3 showed groups of DEGs in common between P1 and T22 samples, showing opposite signs of the gene regulation in the experimental plants. Notably, JA pathway is fundamentally prompted by P1, while in T22, not a single gene associated with this hormonal pathway was retrieved. Similarly, ethylene signaling is stimulated by P1, while it is repressed by T22; SA pathway showed an opposite trend, as can be inferred by the PR-10 transcription level (Table 3).

**Table 3:** Common DEG datasets of tomato treated with *Trichoderma* strains P1 or T22 and grown at 20°C, showing opposite regulation sign.

**Gene ID**	**P1 FC**	**T22 FC**	**Description**
**Oxidativeburst/detoxification**			
Solyc08g074683.1	2.87	−1.31	Polyphenol oxidase precursor
Solyc12g006760.1	−1.52	3.92	Glutathione S–transferase zeta 1
Solyc03g080100.3	−1.16	1.17	Heavy metal transport/detoxification superfamily protein
**JA pathway**			
Solyc04g079730.1	2.72	−1.10	Allene oxide synthase
Solyc12g010030.2	4.17	−1.53	Leucine aminopeptidase
Solyc00g187050.3	3.89	−1.49	Leucine aminopeptidase 2
Solyc07g007250.3	4.62	−1.14	Metallocarboxypeptidase inhibitor
Solyc05g047670.1	2.63	−3.75	Protein Ycf2
**ET pathway**			
Solyc04g077490.3	1.55	−1.04	AP2–like ethylene–responsive transcription factor
Solyc06g053710.3	1.49	−1.19	Ethylene receptor homolog (ETR4)
**SA pathway**			
Solyc07g005370.3	−1.58	1.45	Pathogenesis-related (PR)- protein 10
**Defense-related pathway**			
Solyc02g084850.3	4.43	−2.08	Abscisic acid and environmental stress-inducible protein TAS14
Solyc06g074710.1	4.24	−2.23	Hydroxycinnamoyl-CoAshikimate/quinate hydroxycinnamoyltransferase
Solyc04g049750.3	−1.16	2.56	Pentatricopeptide (PPR) repeat protein
Solyc04g011767.1	−1.14	1.14	Pentatricopeptide repeat-containing protein
Solyc10g085900.2	−1.14	1.1	Tetratricopeptide repeat (TPR)-like superfamily protein
Solyc06g068130.3	1.72	−1.87	Tetratricopeptide repeat (TPR)-like superfamily protein

Tomato genes univocally upregulated or downregulated by P1 or T22 are shown in Supplementary Table 4. *T. atroviride* P1 induced specifically 1,190 transcripts. The transcript with the highest fold change encodes for threonine deaminase, a late defense gene involved in the resistance against chewing insects (Kang et al., 2006; Gonzales-Vigil et al., 2011). Other transcripts coding for proteins with similar functions are those related to leucine aminopeptidase 2, arginase 2, and several classes of proteinase inhibitors. Interestingly, the transcript coding for the hypersensitive response assisting protein is strongly upregulated (Supplementary Table 4A). Several genes involved in ROS scavenging and detoxification are also modulated in their expression (protein detoxification, laccase, NADH dehydrogenase subunit F, superoxide dismutase 1 and 3, and hydrogen peroxide-induced 1). The alteration of early signals in P1 samples is clearly supported by the pathway analysis carried out by MapMan, which identified 79 gene codings for receptor-like kinases, where most of them are upregulated (Supplementary Table 5). In addition, among P1-specific genes are included transcripts coding for several classes of defense-related transcription factors, such as WRKY transcription factor 3, 21, 22, bHLH transcription factor, MYB transcription factor 11, 53, 86, GRAS family transcription factor, zinc finger transcription factor 54, and AP2-like ethylene-responsive transcription factor. Genes univocally downregulated by P1 included S-adenosyl-L-methionine-dependent methyltransferases, S-adenosyl-L-methionine, salicylic acid carboxyl methyltransferase 1, pathogenesis-related protein 1, chitinase, β-glucosidase, defensin, subtilisin, thaumatin, and osmotin (Supplementary Table 4B).

Genes specifically modulated by T22 include 781, and 401 upregulated genes and 380 downregulated genes (Supplementary Tables 4C,D). Functions and ontologies of genes modulated by T22 and P1 are superimposable, despite players are different. T22-specific genes showing high fold-change code for phenylalanine ammonia lyase 4, ethylene-responsive transcription factor, and several heat shock proteins Class I (Supplementary Table 4C). Gene coding for several classes of PR proteins are also induced (chitinase, pathogenesis-related protein 1 and 5), as well as genes involved in SA biosynthesis (phenylalanine ammonia lyase 4 and S-adenosyl-L-methionine-dependent methyltransferases). Ethylene pathway is fundamentally down-represented, since it is observed in the repression of 1-aminocyclopropane-1-carboxylic acid synthase-2, several transcripts coding for ethylene-responsive transcription factor, AP2-like ethylene-responsive transcription factor, and ethylene response factors D2 and 3. A similar trend is observed for the following JA pathway: transcripts coding for lipoxygenase A and D, JA-ZIM domain proteins, and proteinase inhibitors are all downregulated (Supplementary Table 4D).

**Figure 10:**
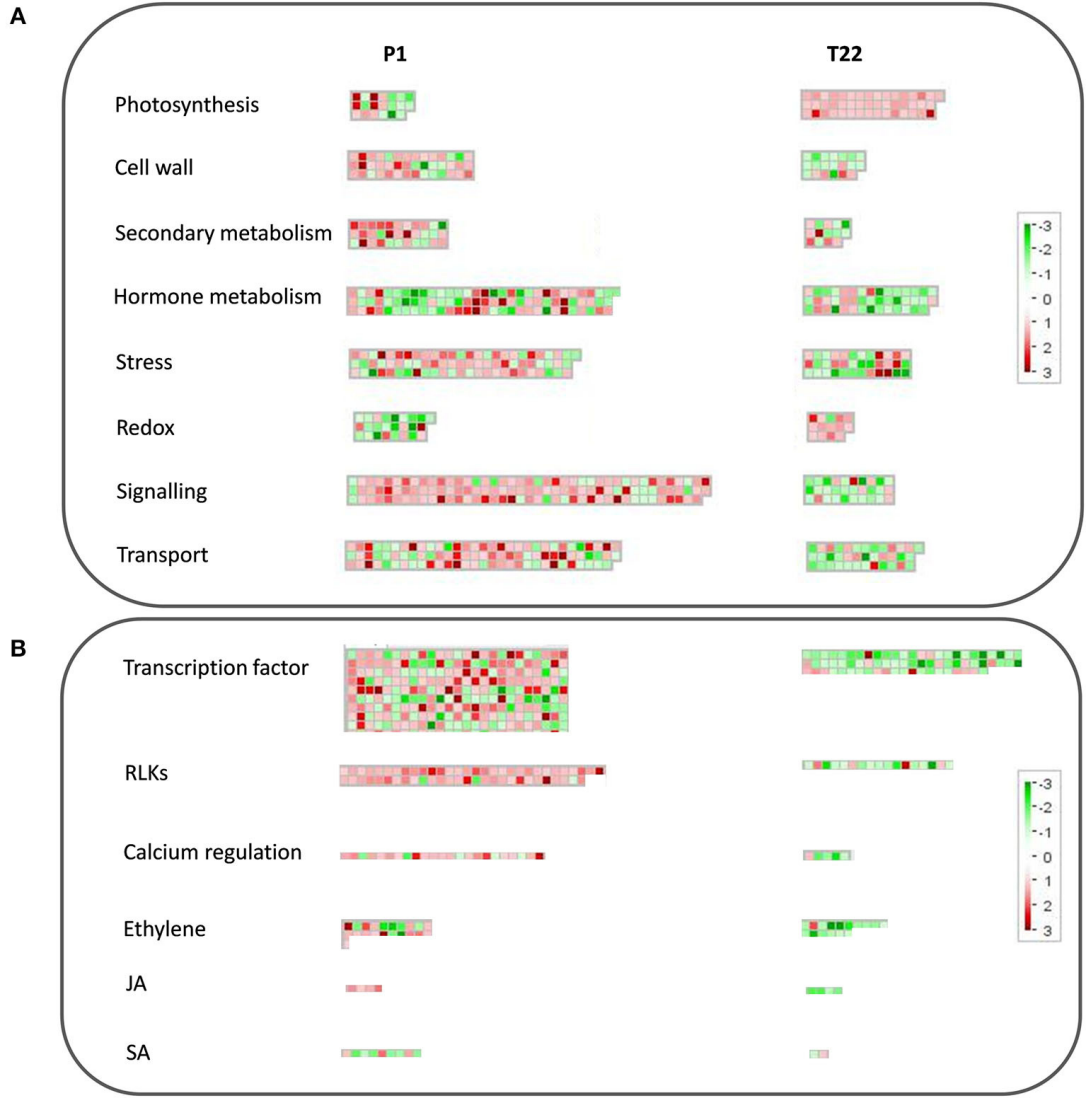
MapMan analysis of tomato transcriptomic profiles imposed by *T. atroviride* P1 or *T. afroharzianum* T22 inocula at 20°C. Organization of tomato by differentially expressed genes (DEGs) in functional categories according to the MapMan ontology across the interaction with spores of P1 or T22. **(A)** Overview of functional categories related to the main cellular processes**; (B)** Overview of the most relevant defense-related regulation processes. Each box represents a transcript; red color indicates upregulation, while green indicates downregulation. Boxes are grouped based on the ontological classification.

An overview of the impact at the cellular level of the used strains of the two *Trichoderma* species is shown in Figure 10. Specific genes of the two interactions are organized in functional categories according to MapMan ontologies (Figure 10). *T. atroviride* P1 showed a stronger impact on tomato transcriptome, affecting the expression of a higher number of genes than *T. afroharzianum* T22. Most of the presented functional categories were induced by P1, while repressed by T22, despite the analysis involves specific genes for both interactions (Figure 10).

### Differential Response of Defense Genes at Different Temperatures During *Trichoderma*-Tomato Interaction

**Figure 11:**
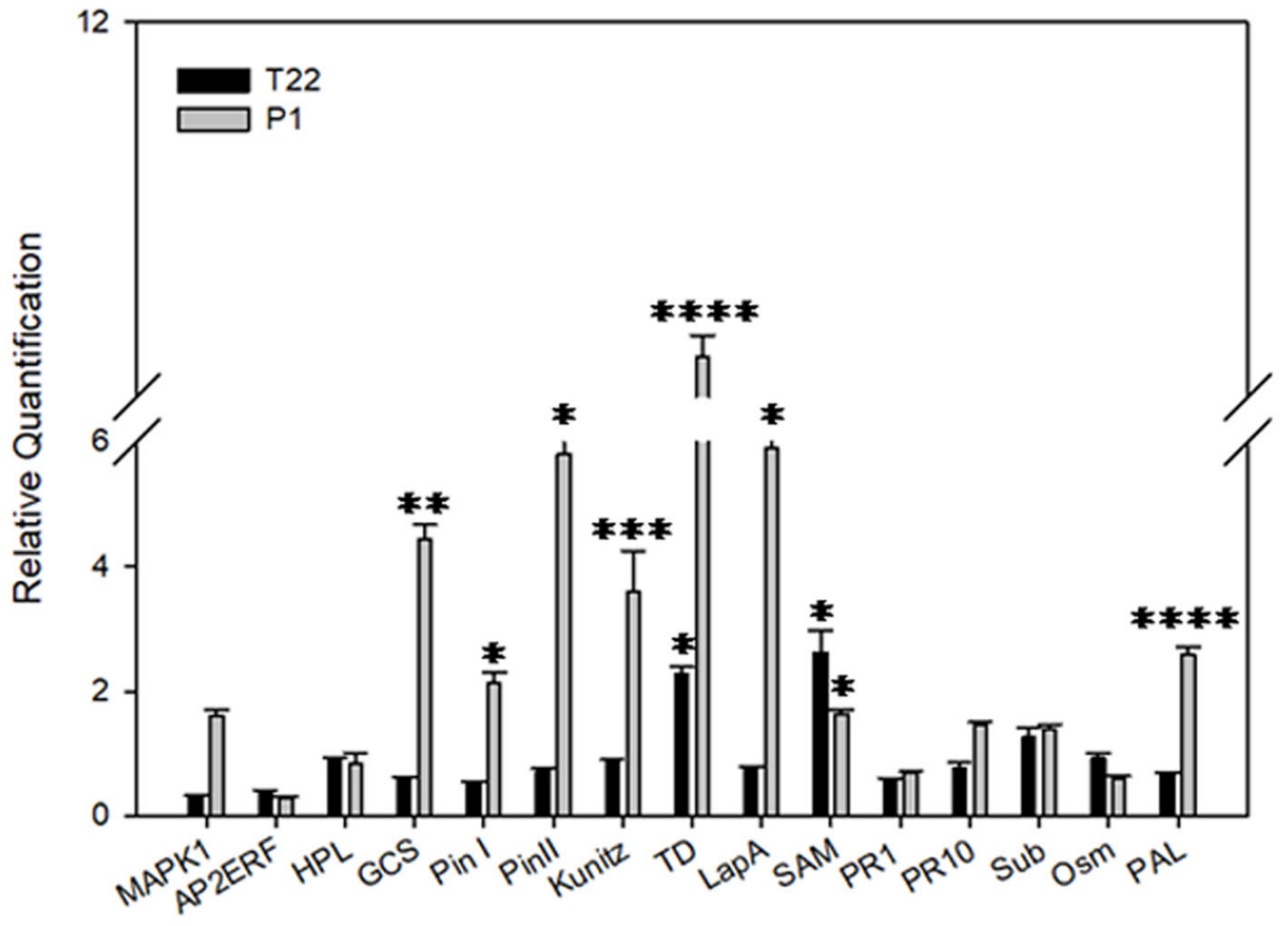
Relative quantification of defense-related genes by real-time PCR (RT-PCR). Relative quantities (RQ) of defense genes in plants treated with *T. afroharzianum* T22 and *T. atroviride* P1 at 20°C. Data are calibrated to the untreated control sample (RQ = 1). Bars represent SE. Asterisks indicate statistically significant differences compared to the control condition (Student's *t*-test, *P < 0.05; **P < 0.01; ***P < 0.001; ****P < 0.0001). *MAPK1*, Map Kinase 1; *AP2ERF*, Ap2 Ethylene responsive factor; *HPL*, hydroperoxide lyase; *GCS*, germacrene C-synthase; *Pin I* and *Pin II*, wound-induced proteinase inhibitor I and II; *Kunitz*, Kunitz type proteinase inhibitor; *TD*, Threonine deaminase; *LapA*, leucine aminopeptidase A; *SAM*, S-adenosyl methionine; *PR1-PR10*, pathogenesis-related proteins 1 and 10; *Sub*, Subtilisin; *Osm*, Osmotin; *PAL*, Phenylalanine ammonia lyase.

In order to assess the combined effect of the *Trichoderma* strain treatments and the environmental temperatures on the interaction with tomato and the subsequent plant defense response, an expression analysis of a selected group of defense genes was carried out. At 20°C, a greater activation of defense genes was almost exclusively noted in the P1-treated plants (Table 3, Figure 11). Nine late defense genes were significantly induced in P1-treated plants when compared to the control (Map Kinase 1, germacrene C-synthase, wound-induced proteinase inhibitor I and II, Kunitz type proteinase inhibitor, threonine deaminase, leucine aminopeptidase A, and phenylalanine ammonia lyase). Instead, in the T22 samples only two genes of threonine deaminase and S-adenosyl methionine were moderately induced in comparison to the control (Figure 11).

**Figure 12:**
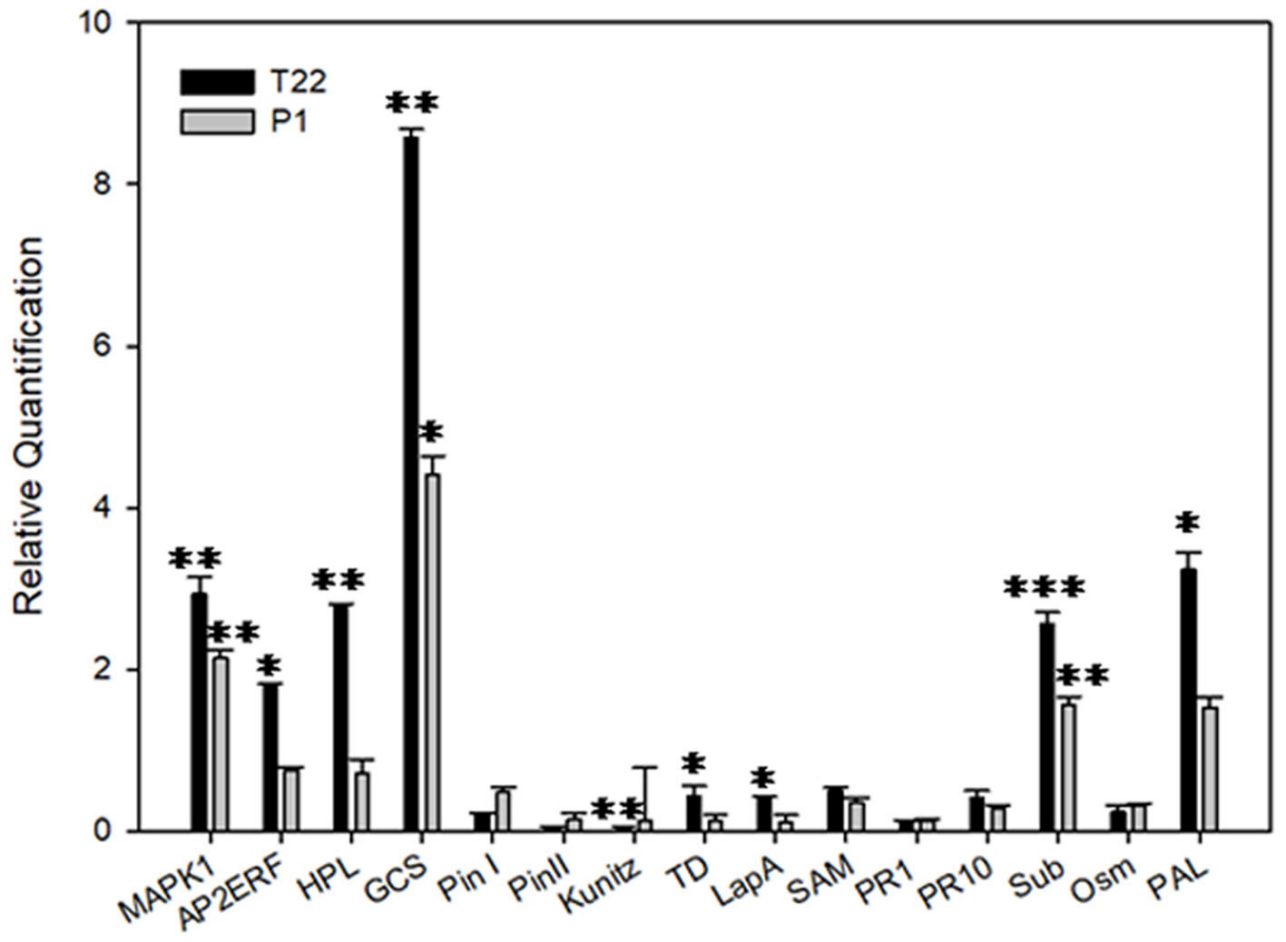
Relative quantification of defense-related genes by real-time PCR (RT-PCR). Relative quantities (RQ) of defense genes in plants treated with *T. afroharzianum* T22 and *T. atroviride* P1 at 25 °C. Data are calibrated to the CTRL sample (RQ = 1). Bars represent SE. Asterisks indicate statistically significant differences compared to the control condition (Student's *t*-test, *P < 0.05; **P < 0.01; ***P < 0.001). *MAPK1*, Map Kinase 1; *AP2ERF*, Ap2 ethylene responsive factor; *HPL*, hydroperoxidelyase; *GCS*, germacrene C-synthase; *Pin I* and *Pin II*, wound-induced proteinase inhibitor I and II; *Kunitz*, Kunitz type proteinase inhibitor; *TD*, threonine deaminase; *LapA*, leucine aminopeptidase A; *SAM*, S-adenosyl methionine; *PR1-PR10*, pathogenesis-related proteins 1 and 10; *Sub*, subtilisin; *Osm*, osmotin; *PAL*, phenylalanine ammonia lyase.

The transcriptional analysis of plants grown at the higher temperature showed an opposite trend for the same gene set, in which the T22 samples were mainly upregulated when compared to the control, even though the regulated genes were slightly different (Figure 12); those genes showing transcriptional rates significantly different from the control included Map Kinase 1, Ap2 ethylene responsive factor, hydroperoxide lyase, germacrene C-synthase, subtilisin, and phenylalanine ammonia lyase.

## Discussion

The use of microorganisms in agriculture as biocontrol agents represents one of the most promising and widespread tools for the sustainable management of plant diseases (Howell, 2003; Harman et al., 2004; Ab Rahman et al., 2018). Beneficial microbes contribute to the improvement of crop quality and yield, concurring to the establishment of useful plant microbiota, which promotes growth, nutrient availability, and resistance against pathogens (Vinale et al., 2008; Castillo Lopez and Sword, 2015; Poveda et al., 2020; Sinno et al., 2020). Numerous microbial strains are components of biological products promoting plant growth and/or inducing disease resistance, and, along with their secondary metabolites may represent very important bioactive components for the development of effective formulations (Hermosa et al., 2013; Rouphael et al., 2015; Woo and Pepe, 2018). Moreover, another possible effect by beneficial microbes is the induced resistance response in the plant, a priming of plant defenses from their interaction that results in a faster and/or a stronger protective response upon subsequent contact or infection by a pathogen (Conrath, 2011; Hermosa et al., 2012; Conrath et al., 2015). Several strains of fungi belonging to the genus *Trichoderma* are not only effective biocontrol agents of plant pathogens, but also stimulators of this priming effect (Lorito et al., 2010).

Despite the continuous research for new formulations, to find effective combinations of microbial strains with different plant species/cultivars, optimal mixtures and doses of microbes plus bioactive molecules to obtain a broad spectrum of biocontrol results on diverse crops, little attention has been dedicated to the environmental abiotic factors that can influence the interaction with the plant.

Temperature is of pivotal importance in the modulation of biological processes, and its connection to geographic location clearly generates spatial heterogeneity that needs to be taken into account when handling living organisms. This aspect is even more relevant if we consider the severe environmental stress associated with global warming and the extreme conditions to which we are increasingly exposed. Climatic changes strongly influence plant physiology and the agroecosystem; thus crop yield and production have an important impact on the world economy. Empirical data indicate that the productivity of many crops worldwide has already declined markedly, as a result of rising temperatures. In the case of maize, for example, global production has declined by 3.8–12.5% over the past three decades (Lobell et al., 2011; Tigchelaar et al., 2018). Considering that global temperatures are forecasted to increase from 2.6 to 4°C before the end of this century (Change, 2014; Rogelj et al., 2016), this trend will have a remarkable impact on the living organisms and their interactions. The severity of the presented scenario does not include other environmental factors/conditions that are predicted to aggravate the situation, that is, the effect of rapid thermic excursions, precipitation and frost, plus CO_2_ release. In this scenario, further studies characterized by an increasing tendency to adopt policy measures (e.g., EU Directive 128/2009) aiming to reduce the use of synthetic pesticides, while promoting integrated pest management (IPM) strategies as a valid alternative, means that plant beneficial microbials are an important tool for a sustainable plant health promotion at a global level. However, their profitable use requires an in-depth understanding of the effects that climatic conditions may have on their efficiency in controlling pests and disease agents, in order to select the most appropriate strains to a specific environment.

To address this goal, we studied the endogenous defenses of tomato plants as affected by two specific *Trichoderma* species strains. It is expected that *T. atroviride* P1 should have a better performance at lower temperatures, given its original reasons for selection (Tronsmo, 1989) and the profitable use for post-harvest protection in cold storage (Tronsmo, 1991). The beneficial effects of these fungi on tomato plants have been already reported (Harman et al., 2004; Tucci et al., 2011; Nandini et al., 2017; Chen et al., 2019; Coppola et al., 2019a,b), but without special consideration to the effect of temperature, which may have an important impact both on fungal development and on the plant-*Trichoderma* interactions which may be relevant for crop protection.

Different growth was observed for *T. afroharzianum* T22 and *T. atroviride* P1 at 20 and 25°C, both in *in vitro* and *in vivo* test conditions. The lower temperature (20°C) fostered P1 growth and its capacity to colonize tomato plants, whereas T22 germinated and grew more abundantly at the higher temperature (25°C). This is well in line with available experimental evidence supporting that *T. atroviride* P1 is a cold-adapted isolate (Hjeljord et al., 2000), and corroborating the importance of temperature in modulating the fungal development, as already reported for other *T. atroviride* strains (Daryaei, 2014; Daryaei et al., 2016).

The difference in fungal growth observed in the present study, along with data on the induction of insect resistance and the underlying transcriptome reprogramming, well account for the temperature-dependent biocontrol activity observed for the two fungal strains considered. Based on our previous transcriptomic data from experiments with Dwarf San Marzano tomato performed at 20°C and treatments with *T. atroviride* P1 (Coppola et al., 2019a) and *T. afroharzianum* T22 (Coppola et al., 2019b), we were able to choose a representative gene set involved in defense-related pathways and analyze their transcriptional profile by qRT-PCR, by comparing the two plant-fungus associations to determine any temperature-dependent effects.

The present findings were consistent with the expected beneficial effects of *Trichoderma* on the activation of tomato endogenous defense processes: the upregulation of transcripts coding for several families of transcription factors and genes associated with oxidative burst and involved in ethylene signaling are indicative of the instauration of a primed state (Conrath, 2011; Broekgaarden et al., 2015; González-Bosch, 2018). A common gene upregulated in the T22- or P1-treated tomato, encodes for purple acid phosphatases 17 (PAPs17), an enzyme that is involved in the plant regulation of phosphorous uptake but also contributes to other biological functions including peroxidation, ascorbate recycling, mediation of salt tolerance, and regulation of cell wall carbohydrate biosynthesis (Ravichandran et al., 2013). PAPs carry predicted signal peptides and, presumably, are secreted; however, the biological function of these proteins in the extracellular space is unknown (Kaffarnik et al., 2009). The *Arabidopsis* PAP5 is involved in the basal resistance against several plant pathogens (Ravichandran et al., 2013) and its optimal level is crucial for mounting complete basal resistance against pathogens (Ravichandran et al., 2015).

The activation of multiple responses may be derived from the action of several *Trichoderma* elicitors that induce resistance *via* different parallel signaling pathways (Hermosa et al., 2013). The interaction of *Trichoderma* (P1 or T22) with tomato could be responsible for the downregulation of genes related to plant cell wall synthesis: the fungus secretes endoglucanases and other cellulolytic enzymes in order to invade and colonize plant tissues (Klose et al., 2015; Sonoda et al., 2019). Both datasets from tomato treated with either of the *Trichoderma* demonstrated a repression of transcripts encoding for enzymes involved in the process of vegetative cell wall reconstruction that may reflect a strategy shared by these fungi to colonize the plant.

P1-treated plants showed upregulated genes related to JA production, which supports the negative performance on the moth larval growth, development, and adult reproduction. On the contrary, the JA as well as the ethylene pathway was not induced in T22-treated plants, while the SA pathway was induced, as shown by the upregulation of genes involved in both synthesis and signaling of this plant hormone. This was consistent with evidence from bioassays with the aphids, in which their survival and reproduction were significantly impaired when plants interacted with the fungus. Aphid susceptibility to defensive pathways controlled by different plant hormones has been a matter of debate (Avila et al., 2012; Studham and MacIntosh, 2013; Duhlian et al., 2020). Both synergistic and inhibitory aspects of the cross-talk among JA, SA, and ET pathways have been reported (Morkunas and Gabryś, 2011), a complex scenario in which the role of aphid effectors and suppressors of host-defense responses make the comprehension very difficult (Escudero-Martinez et al., 2020). Thus, the reduction of aphid survival observed in P1-treated plants at the low temperature appears to be associated with the upregulation of a wide group of ethylene-related genes, which is different from that observed in the T22-treated plants. Several studies have examined the role of ethylene in plant–aphid interactions, and aphids have been shown to induce an ethylene burst in several different plant species (Miller et al., 1994; Argandona et al., 2001; Mantelin et al., 2009). Moreover, the role of ET against aphids has been separated by the influence of JA; for instance, Louis et al. (2015) demonstrated a subtle hormonal equilibrium between JA- and ET-enabled maize to respond differentially against chewing and phloem-feeding insects. The maize insect resistance 1 (mir1) gene product, a cysteine (Cys) proteinase that is a maize key defensive protein, is induced by JA-ET and active against chewers. Mir1-mediated resistance to corn leaf aphid (CLA; *Rhopalosiphum maidis*) is independent of JA but regulated by the ET-signaling pathway (Louis et al., 2015). These findings are consistent with the evidence collected on P1 plants ascribable exclusively to ET.

Interestingly, the relative expression of selected plant defense genes clearly mirrors the performance of the insect at different temperatures. In fact, at lower temperatures, P1-tomato plants have a more pronounced negative impact on *Spodoptera* fitness, compared to the T22-tomato plants. These effects are likely induced by the observed higher expression of genes coding for enzymes that interfere with protein digestion/assimilation in insects. Similarly, the negative impact of P1-tomato plants on the longevity of aphids at low temperature can be due to the enhanced transcription rate of *Phenylalanine ammonia lyase* (*PAL*) gene, coding for a key enzyme in the biosynthesis of salicylic acid that is involved in the defense against aphids and pathogens. This conclusion was corroborated by the reverse results observed with the two *Trichoderma* at higher temperature in terms of control efficacy, as supported by a similar gene expression profile, characterized by opposite trends between P1 vs. T22. Indeed, *PAL* is upregulated in T22-tomato plants (in addition to the PR protein, subtilisin), accounting for the higher resistance against aphids, while the lower performance of *S. littoralis* was associated with the disruption of digestion induced by a moderate induction of transcripts coding for several defense molecules controlled by the jasmonate pathway.

Taken into consideration all of our data, the results demonstrated that a better performance of *T. atroviride* P1-tomato interaction occurred at 20°C, potentiating the native defense tools of the cultivar Dwarf San Marzano: active against Lepidopteran and aphids *via* the enhancement of JA- and/or ET-mediated responses. Conversely, *T. afroharzianum* T22 reached its best performance at 25°C, promoting plant endogenous defenses mainly mediated by SA. The targeted effect of root colonization of *Trichoderma* species could be an interesting consideration in a future investigation.

In conclusion, our results clearly indicate that the outcome of a plant–*Trichoderma* interaction is strongly influenced by temperature; such information is most useful in allowing a rational selection of species/strain, better adapted to different climatic zones and to local environmental conditions, for a more effective and predictable use of these important biocontrol agents.

## Data Availability Statement

The datasets presented in this study can be found in online repositories. The names of the repository/repositories and accession number(s) can be found in the article/Supplementary Material.

## Ethics Statement

Animal subjects involved in the study were insect pests (aphids and caterpillars) that we manage on agricultural crops using biocontrol agents (BCA).

## Author Contributions

MCD, FP, RR, SLW, and ML contributed to the study design. MC, EC, DM, and ID performed the experiments and analyzed the results. MCD, SLW, and RR supervised the experiments. MC and MCD wrote the first draft of the manuscript. SLW, RR, and FP revised the first draft and wrote new sections. All authors contributed to revise the manuscript, read, and approved the submitted version.

## Conflict of Interest

The authors declare that the research was conducted in the absence of any commercial or financial relationships that could be construed as a potential conflict of interest.
