# Supplementary material for: Temperature Differentially Influences the Capacity of Trichoderma Species to Induce Plant Defense Responses in Tomato Against Insect Pests
Source: Front Plant Sci. 2021 Jun 9;12:678830. doi: 10.3389/fpls.2021.678830 (PMC8221184; doi:10.3389/fpls.2021.678830)
Supplement: Supplementary file 1 [file Data_Sheet_1.zip › Supplementary Figure 1.DOCX]

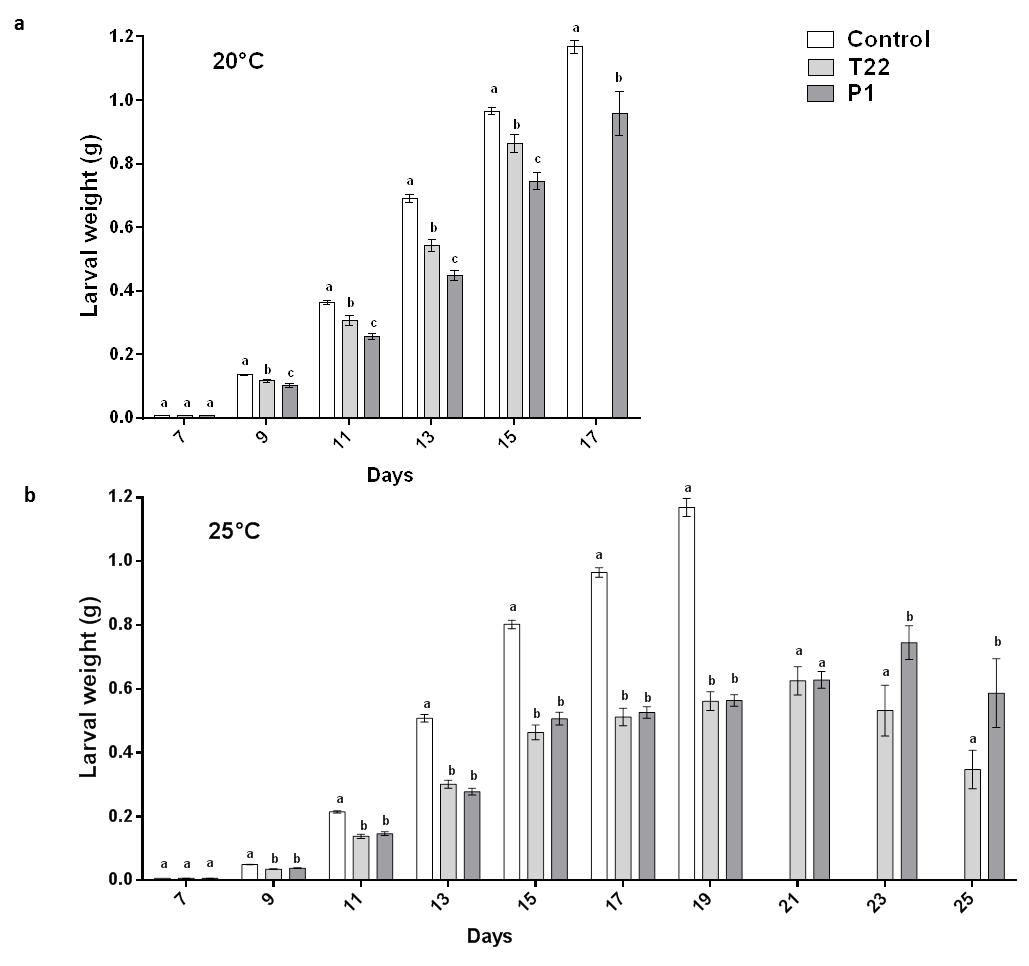


**Supplementary Figure 1**

***Spodoptera littoralis* larvae weight.** Experimental larvae, fed leaves from tomato plants inoculated with *Trichoderma* strain T22 or P1, then grown at 20°C (a), or 25°C (b)**.** The values are means ± standard errors. In a single day, different letters indicate a significant difference (P<0.05). Missing values on both graphs indicates the onset of pupation for that sample. Mean values within each sampling occasion denoted with different letters are significantly different. Punctual statistical indices are reported in Supplementary Table 2.
