# Supplementary material for: Temperature Differentially Influences the Capacity of Trichoderma Species to Induce Plant Defense Responses in Tomato Against Insect Pests
Source: Front Plant Sci. 2021 Jun 9;12:678830. doi: 10.3389/fpls.2021.678830 (PMC8221184; doi:10.3389/fpls.2021.678830)
Supplement: Supplementary file 1 [file Data_Sheet_1.zip › Supplementary Figure 2.DOCX]

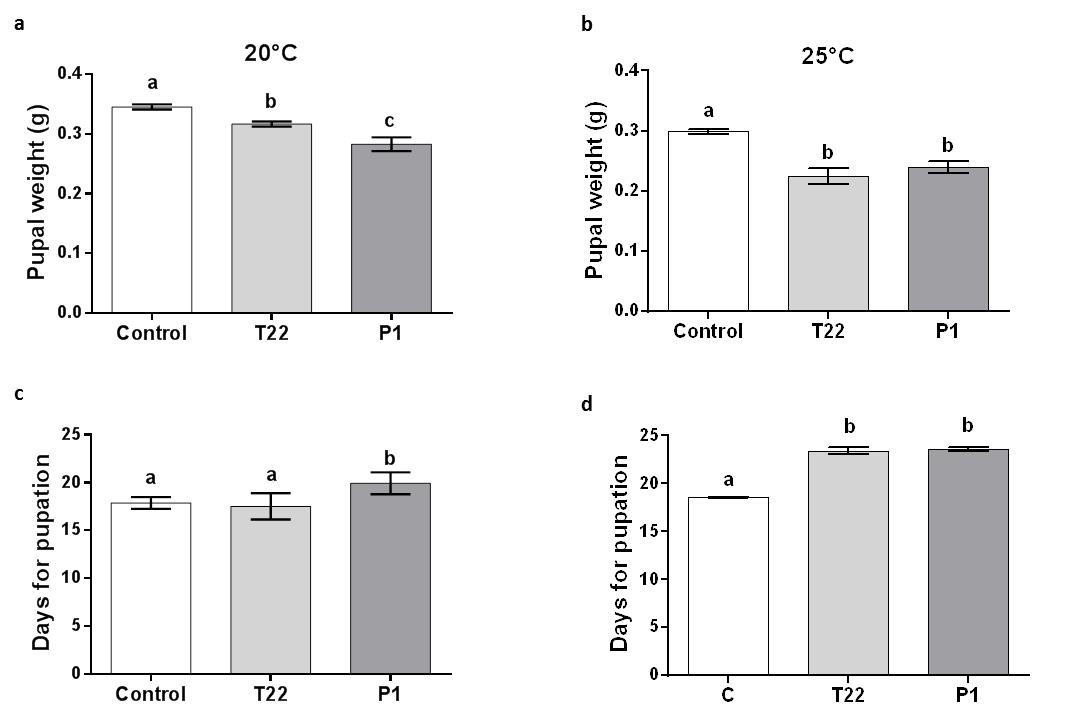


**Supplementary Figure 2**

***Spodoptera littoralis* pupal weight and days required for pupation** when larvae were fed leaves from tomato plants inoculated with *Trichoderma* strain T22 or P1 then grown at 20°C (a and c), or 25°C (b and d). The values are means ± standard errors. Different letters indicate a significant difference (Kruskal-Wallis test, P<0.05). Statistical indices for pupal weights: at 20°C, Kruskal-Wallis test: KW = 29, P < 0.0001 (a); at 25°C, KW=31.36 P< 0.0001 (b). Statistical indices for time for pupation: at 20°C, KW=46.46, P< 0.0001 (c); at 25°C, Kruskal-Wallis test: KW = 57.59, P < 0.0001 (d).
