# Supplementary material for: Temperature Differentially Influences the Capacity of Trichoderma Species to Induce Plant Defense Responses in Tomato Against Insect Pests
Source: Front Plant Sci. 2021 Jun 9;12:678830. doi: 10.3389/fpls.2021.678830 (PMC8221184; doi:10.3389/fpls.2021.678830)
Supplement: Supplementary file 1 [file Data_Sheet_1.zip › Supplementary Table 1.DOCX]

Supplementary table 1. Primer used for the expression analysis by Real Time RT-PCR and their mail features. Table lists primer name, sequence, gene name and identifier (Solgenomics network).

| Primer name | Primer sequence 5'-3' | Gene name | Gene ID |
| --- | --- | --- | --- |
| Ef Fw | CTCCATTGGGTCGTTTTGCT | Elongation factor 1a | X53043 |
| Ef Rv | GGTCACCTTGGCACCAGTTG |  |  |
| MAPK1 Fw | TTTTGATTGTCGGAATGCCG | MAP Kinase 1 | Solyc12g019460.1 |
| MAPK1 Rv | CCTCCAGTACATTCTCCGACCA |  |  |
| PR1 Fw | ATGCAACACTCGGTGGACCTT | Pathogenesis-related protein 1 | Solyc00g174340.1 |
| PR1 Rv | CCATTGCTTCTCATCAACCCA |  |  |
| AP2 ERF Fw | GCCATTTGATAACACCGCCC | AP2-ERF Transcription factor | Solyc11g010710.1.1 |
| AP2 ERF Rv | TGGTTGTAGCGTTGGAAGCA |  |  |
| HPL Fw | AGCATTGGTGTCCTTCAACC | Hydroperoxide lyase (HPL) | Solyc07g049690.2.1 |
| HPL Rv | CGTTTTCTGGATTTACGCCGA |  |  |
| Kunitz Fw | TTGTTGGAGACGGAAGGAAGC | Kunitz-type proteinase inhibitor 1 | X73986.1 |
| Kunitz Rv | CGGCAAAATGGACAAAGCAC |  |  |
| OSM Fw | AGTTGGTGGTTTTGGGCTCC | Osmotin-like protein | Solyc08g080670.1.1 |
| OSM Rv | AGACTCCACCACAATCACCG |  |  |
| Pin I Fw | GAAACTCTCATGGCACGAAAAG | Proteinase inhibitor I | K03290 |
| Pin I Rv | CACCAATAAGTTCTGGCCACAT |  |  |
| Pin II Fw | CCAAAAAGGCCAAATGCTTG | Proteinase inhibitor II | K03291 |
| Pin II Rv | TGTGCAACACGTGGTACATCC |  |  |
| Sub Fw | CTCCCACGGTCACCATAACA | Subtilisin | Solyc01g087840.2.1 |
| Sub Rv | ATCTGGCTTTGCGATTCCCA |  |  |
| Lap Fw | ATCTCAGGTTTCCTGGTGGAAGGA | Leucine aminopeptidase | U50152 |
| Lap Rv | AGTTGCTATGGCAGAGGCAGAG |  |  |
| TD Fw | TTAGACGCTTTCTCCCCTCGT | Treonine deaminase | M61914 |
| TD Rv | GCTTGAGGAACTTGGAATCCC |  |  |
| PR10 Fw | CATCATGTGACCACGAATGGA | Pathogenesis-related protein 10 | AK329477 |
| PR10 Rv | AACGTGAAGGACAAAACCCAAG |  |  |
| GCS Fw | TTGGTGAAGCCTTAACTCAGCC | Germacrene-C-synthase | AF279454.1 |
| GCS Rv | GCAAATGGTGGTGTGCATCAT |  |  |
| PAL Fw | CCACATTCAGCAACAAGGGC | Phenylalanine ammonia lyase | Solyc09g007890.1.1 |
| PAL Rv | ACACGGGGTAATGTTGCTGT |  |  |
| SAM Fw | TGTGCCAGTGTATTTTCCCTCT | S-adenosyl-L-methionine salicylic acid carboxyl methyltransferase | Solyc01g005230.2.1 |
| SAM Rv | CATCAGCCTCATCCACAAGT |  |  |
