# Supplementary material for: Temperature Differentially Influences the Capacity of Trichoderma Species to Induce Plant Defense Responses in Tomato Against Insect Pests
Source: Front Plant Sci. 2021 Jun 9;12:678830. doi: 10.3389/fpls.2021.678830 (PMC8221184; doi:10.3389/fpls.2021.678830)
Supplement: Supplementary file 1 [file Data_Sheet_1.zip › Supplementary Table 2.DOCX]

**Supplementary Table 2**. Values obtained from the statistical analysis comparing of *Spodoptera littoralis* larvae weight following feeding of larvae on tomato leaves from plants treated with Trichoderma P1 or T22 and grown at 20°C or 25°C, as measured at different time points (days) (see Suppl. Fig. 1).

| **Day** | **20°C** | **25°C** |
| --- | --- | --- |
| **7** | One Way ANOVA: F_(2,93)_ = 0.8594; P = 0.4268 | One Way ANOVA: F_(2, 93)_ = 1.283; P = 0.2822 |
| **9** | Kruskal-Wallis test: KW = 20.79; P < 0.0001 * | One Way ANOVA: F_(2, 93)_ = 46.80; P < 0.0001* |
| **11** | Kruskal-Wallis test: KW = 19.55; P < 0.0001 * | Kruskal-Wallis test: KW = 53.85; P < 0.0001* |
| **13** | One Way ANOVA: F_(2,90)_ = 30.667; P < 0.0001 * | One Way ANOVA: F_(2, 91)_ = 114.6; P < 0.0001* |
| **15** | Kruskal-Wallis test: KW = 44.51; P < 0.0001 * | Kruskal-Wallis test: KW = 62.36; P < 0.0001* |
| **17** | Student’s t test: t = 2.246 df = 19; P = 0.00368 | One Way ANOVA: F_(2, 89)_ = 155.9; P < 0.0001* |
| **19** |  | Kruskal-Wallis test: KW = 36.9; P < 0.0001* |
| **21** |  | Student’s t test: t = 0.1022; df = 45; P = 0.919 |
| **23** |  | Student’s t test: t = 2.321; df = 29; P = 0.275 |
| **25** |  | Student’s t test: t = 1.276; df = 8; P = 0.02377* |
